# Cerebral arterial pulsatility is associated with features of small vessel disease in patients with acute stroke and TIA: a 4D flow MRI study

**DOI:** 10.1007/s00415-019-09620-6

**Published:** 2019-11-14

**Authors:** Johan Birnefeld, Anders Wåhlin, Anders Eklund, Jan Malm

**Affiliations:** 1 Department of Pharmacology and Clinical Neuroscience, Umeå University, 90187 Umeå, Sweden; 2 Department of Radiation Sciences, Umeå University, Umeå, Sweden; 3 Umeå Centre for Functional Brain Imaging, Umeå University, Umeå, Sweden; 4 Centre for Biomedical Engineering and Physics, Umeå University, Umeå, Sweden

**Keywords:** 4D flow MRI, Pulsatile index, Pulsatility, Small vessel disease, White matter hyperintensities

## Abstract

Cerebral small vessel disease (SVD) is a major cause of stroke and cognitive impairment. However, the underlying mechanisms behind SVD are still poorly understood. High cerebral arterial pulsatility has been suggested as a possible cause of SVD. In population studies, arterial pulsatility has been linked to white matter hyperintensities (WMH), cerebral atrophy, and cognitive impairment, all features of SVD. In stroke, pulsatility data are scarce and contradictory. The aim of this study was to investigate the relationship between arterial pulsatility and SVD in stroke patients. With a cross-sectional design, 89 patients with acute ischemic stroke or TIA were examined with MRI. A neuropsychological assessment was performed 1 year later. Using 4D flow MRI, pulsatile indices (PI) were calculated for the internal carotid artery (ICA) and middle cerebral artery (M1, M3). Flow volume pulsatility (FVP), a measure corresponding to the cyclic expansion of the arterial tree, was calculated for the same locations. These parameters were assessed for associations with WMH volume, brain volume and cognitive function. ICA-FVP was associated with WMH volume (*β* = 1.67, 95% CI: [0.1, 3.24], *p* = 0.037). M1-PI and M1-FVP were associated with decreasing cognitive function (*β* = − 4.4, 95% CI: [− 7.7, − 1.1], *p* = 0.009 and *β* = − 13.15, 95% CI: [− 24.26, − 2.04], *p* = 0.02 respectively). In summary, this supports an association between arterial pulsatility and SVD in stroke patients, and provides a potential target for further research and preventative treatment. FVP may become a useful biomarker for assessing pulsatile stress with PCMRI and 4D flow MRI.

## Introduction

Cerebral small vessel disease (SVD) is a major cause of stroke and cognitive impairment. It is estimated that 20–42% of ischemic strokes are of small vessel origin [1], and conversely, patients with stroke have a higher burden of SVD compared to the population at large [2]. In addition, stroke patients with SVD have worse functional and cognitive outcomes [3, 4]. Radiologically, SVD is characterized by white matter hyperintensities (WMH), lacunar infarctions, cerebral atrophy, cerebral microbleeds, and enlarged perivascular spaces [5]. However, the underlying causes of SVD are poorly understood. Pulsatile stress, persisting over long time, has been suggested as a possible cause of SVD [6, 7]. The aorta is a compliant artery, which under normal conditions dampens the cardiac-driven pulsatility through the Windkessel effect [8]. With age and disease, however, the aorta stiffens which over time may enable for gradually increasing pulsations reaching the distal cerebral arteries and microvasculature [6, 7]. In population studies, increased cerebral arterial pulsatility, represented by Gosling’s Pulsatility Index (PI) [9], has been linked to white matter hyperintensities [10, 11], cerebral atrophy [12, 13], and cognitive impairment [12]. However, data regarding the relationship between pulsatility, WMH [14–16], and cognitive impairment [17] in stroke patients are more scarce and somewhat contradictory. The relationship between pulsatility and cerebral atrophy in stroke patients has not been studied. This calls for a comprehensive study of the relationship between cerebral arterial pulsatility and WMH, cerebral atrophy, and cognitive impairment in stroke.

A new promising technique able to quantify cerebral arterial pulsatility is 4D flow MRI, which measures blood flow in three dimensions time-resolved over the cardiac cycle. The technique measures blood flow rate in any cerebral blood vessel down to a diameter of a few millimeters [18]. In addition to PI, 4D flow MRI is able to measure flow volume pulsatility (FVP). This is an estimation of the downstream arterial tree’s volume expansion with each heartbeat and could be a more physiological way to approach cerebral pulsatility.

We hypothesized that in stroke, high cerebral arterial pulsatility would be associated with more WMH, cerebral atrophy, and cognitive impairment. The aim of this study was to investigate the cross-sectional relationship between arterial pulsatility and SVD in patients with ischemic stroke and transient ischemic attack (TIA).

## Materials and methods

Eighty-nine patients with acute ischemic stroke or TIA were divided in two groups: with or without associated SVD. MRI including 4D flow MRI was performed within the first week and neuropsychological tests 1 year after the event (*n* = 48). Pulsatility measures in proximal and distal cerebral arteries were analyzed for correlations to three main outcomes: WMH volume, total brain volume (TBV), and neuropsychology and adjusted for risk factors.

### Study population

Within a larger project, we prospectively recruited consecutive patients, aged 18–85 years, admitted to the stroke unit at Umeå University with ischemic stroke, TIA, or carotid artery stenosis (CAS) during 2013–2015. Due to fire safety regulations at the research MRI facility, bedridden patients were not included. Exclusion criteria included previous stroke ipsilateral to the current event, remaining disability from a previous stroke (or modified Rankin scale > 1 for any reason) and previous disease of the central nervous system. In addition, patients with any MRI contraindication, systolic blood pressure (SBP) > 180 mmHg, mini-mental state examination < 23 points or inability to understand oral information and/or provide informed consent were excluded. In total, 157 patients were included.

At initial presentation, a clinical evaluation was performed by a stroke physician including medical history, cardiopulmonary- and neurological examination, National Institutes of Health Stroke Scale (NIHSS), standard blood tests, ECG, computed tomography of the brain (CT) and CT angiography (CTA), or cervical ultrasonography. Use of antihypertensive medications were recorded by class (beta blockers, RAAS inhibitors, calcium channel blockers, and diuretics). Examination with Holter monitor and transthoracic- or transesophageal echocardiography was performed on clinical indication. MRI studies were performed at mean 4 (3) days after symptom onset. Finally, an etiologic classification according to TOAST was performed using a validated, computerized tool [19–22].

From the initially included (*n* = 157), we excluded patients aged below 50 years (*n* = 7) with CAS > 70% (*n* = 24) and patients without stroke or TIA within the last 7 days (*n* = 5). Among remaining patients (*n* = 121), 17 did not have a complete MRI examination and a further 15 were excluded for technical reasons, such as motion artefacts or corrupt data. The final study population thus contained 89 patients.

### Neuropsychological testing

Approximately 1 year after the initial examination (354 (27) days), patients were offered a follow-up examination including neuropsychological testing. This later date was selected to avoid acute, reversible effects from the cerebrovascular event. Forty-eight (53.9%) patients participated. Patients with vascular cognitive impairment typically present with frontal lobe deficits such as impaired executive functions, psychomotor speed, and attention [23]. With this in mind, we selected a validated neuropsychological test battery [24, 25] including the following subtests: two choice reaction, Stroop congruent and -incongruent, ten word list, delayed recall, delayed recognition, and trail making tests A and B. Each patient test result was converted to a *Z*-score. An aggregate score was then created by summing the *Z*-score for each test. The test battery is entirely computerized and largely self-instructing and self-evaluating. The test was supervised by a research nurse blinded to pulsatility, WMH and TBV data but not to stroke size and location.

### Brain imaging

MRI studies were performed at a 3 T scanner (Discovery MR 750; GE Healthcare, Milwaukee, Wisconsin) with a 32-channel head coil. T1- and T2-weighted, FLAIR, diffusion- and susceptibility-weighted images were obtained. 4D flow MRI data were acquired by a balanced five-point PC-VIPR sequence [18, 26], covering the intracranial space. The following parameters were used: 16,000 radial projections; acquisition resolution, 300 × 300 × 300; imaging volume, 22 × 22 × 22 cm; reconstruction resolution, 320 × 320 × 320 mm (zero padded interpolation); velocity encoding, 110 cm s^−1^; TR/TE, 6.5/2.7 ms; flip angle, 8°. Dynamic images were reconstructed by retrospective gating from the recorded peripheral pulse signal by use of temporal interpolation similar to view sharing in Cartesian acquisitions. Besides 3D velocity information for the 20 reconstructed time positions, a time-average composite complex difference image was reconstructed to provide an angiogram.

### White matter hyperintensities and brain volume

Using SPM 12 (Wellcome Trust Centre for Neuroimaging, London, UK), the intracranial space was segmented into grey matter (GM), white matter (WM), and cerebrospinal fluid (CSF). Results were visually inspected for accuracy; no manual corrections were needed. Intracranial volume was calculated by summing GM, WM, and CSF. Total brain volume was calculated by summing GM and WM.

WMH volumes were segmented by the lesion prediction algorithm as implemented in the Lesion Segmentation Toolbox version 2.0.15 (https://www.statistical-modelling.de/lst.html) for SPM [27]. Using FreeView (version 1.0, the General Hospital Corporation, Boston, MA), volumes were then visually inspected for accuracy and corrected as needed by an operator blinded to pulsatility data. Corrections were limited to excluding lesions with high DWI signal, interpreted as acute infarctions, and extracerebral features such as the cerebral falx and transverse sinus. In all tissue segmentations, lacunes were considered CSF. WMH volume and TBV were expressed as %ICV.

### Small vessel disease and lesion locations

Structural MRI data were reviewed for lesion sizes, locations, and features of SVD. Visual rating of WMH according to the Fazekas scale was performed [28]. To assess reliability a second, independent reviewer also rated all cases. Ratings were performed blinded to pulsatility data. Interrater agreement (ICC, two-way random, single rater, absolute agreement) was 0.86 (95% CI: [0.65, 0.93]), which was considered good. As expected, there was a strong correlation between Fazekas score and WMH volume (*R* = 0.87; *p* < 0.001). The STRIVE definition of SVD on conventional MRI includes white matter hyperintensities, small recent subcortical infarcts, lacunes, and cerebral microbleeds [5]. For the purposes of this study, SVD was defined as a composite of either Fazekas score ≥ 4, presence of small recent subcortical infarct, lacune, or > 2 cerebral microbleeds, all in turn defined according to STRIVE. Acute lesions were classified according to location as either cortical, subcortical, mixed cortical/subcortical, infratentorial, or no lesion. All visual ratings were performed blinded to pulsatility data.

### Cerebral blood flow

**Fig. 1:**
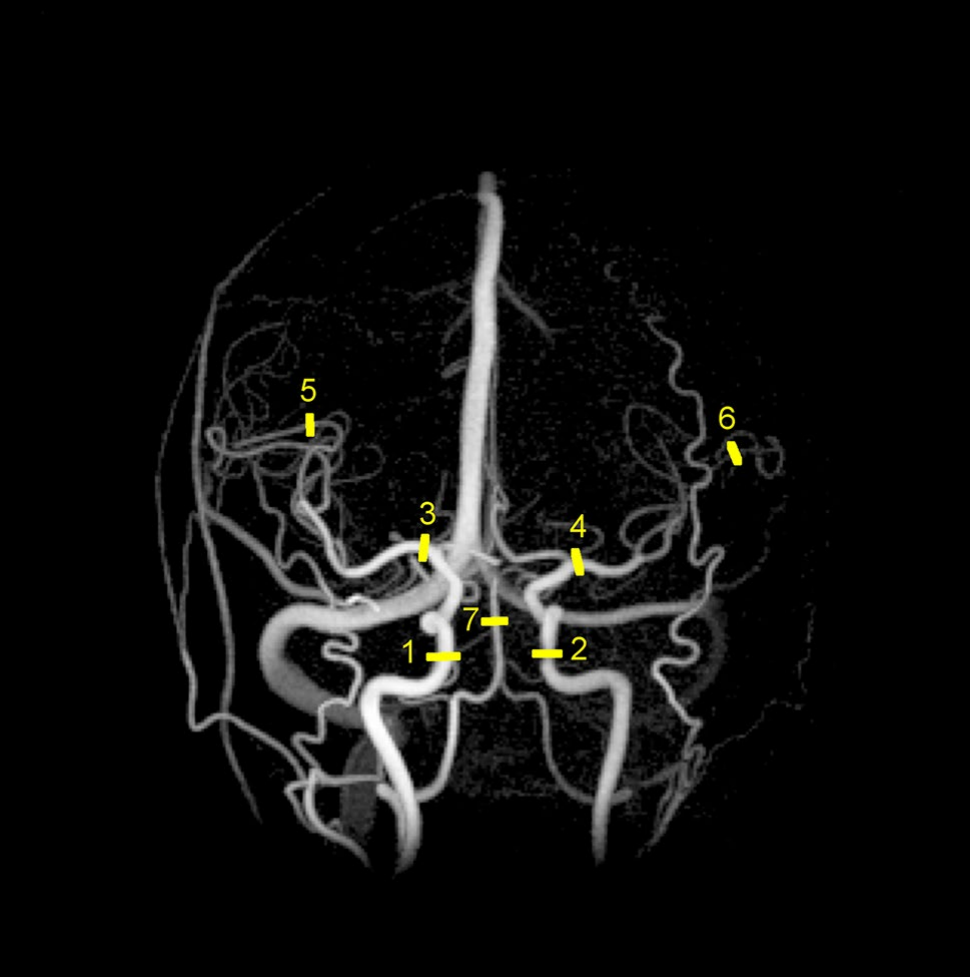
Angiogram generated from PC-VIPR complex difference image. Cerebral arterial pulsatility was measured in the cavernous segment of the internal carotid arteries (1, 2) and in the M1- (3, 4) and M3 (5, 6) segments of the middle cerebral arteries

**Fig. 2:**
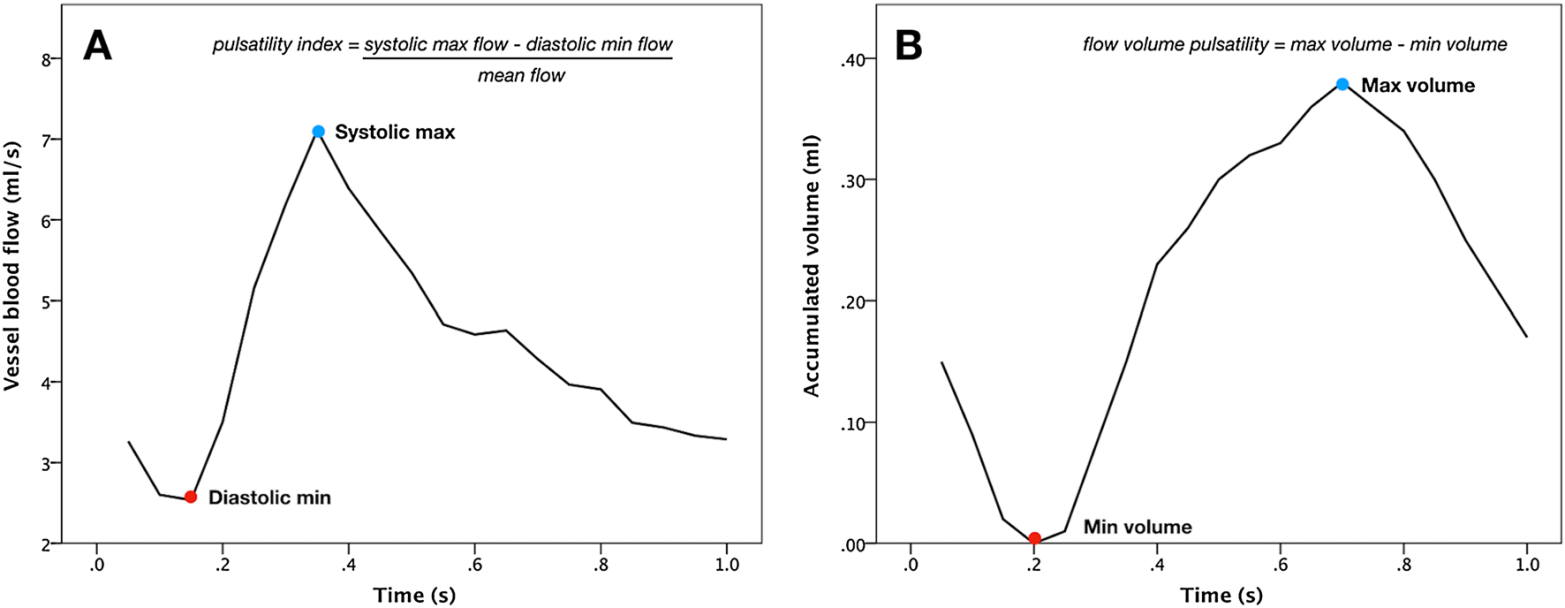
Example waveforms from internal carotid artery. **a** Pulsatility index was calculated as the difference between systolic maximum and diastolic minimum flow rates divided by the mean flow rate. **b** Flow volume pulsatility (FVP) was calculated as the cumulative integral of the blood flow rate waveform after subtraction of the mean flow rate. FVP was defined as the difference between the systolic maximum and the diastolic minimum volumes. The curve has been shifted vertically, so that minimum volume corresponds to zero. Both measures were further standardized by *R*–*R* interval

To assess blood flow in the 4D flow MRI data, a centerline processing scheme was used. It has been previously described in detail [29]. Total cerebral blood flow (tCBF) was measured by summing the mean flow rate of the right and left internal carotid arteries (ICA) and the basilar artery (BA). This excludes flow to the PICAs and AICAs. However, these are small arteries and any effects from omitting this flow were deemed negligible. Pulsatility was measured in the cavernous segment of ICA and in the M1 and M3 segments of the middle cerebral artery (MCA) (Fig. 1). Measurements were made as an average of six consecutive centerline voxels. This frequently corresponds to the length of the M1 segment proximal to its first bifurcation. Thus, in the name of consistency, this was selected as the length of the sampled segments at all locations. PI was calculated for all arteries by dividing the difference between the systolic and diastolic flow rates with the mean flow rate (Fig. 2). FVP was calculated from the cumulative integral of the flow rate waveform after subtraction of the mean flow rate. FVP was defined as the difference between the maximum and minimum volumes (Fig. 2) [26, 30, 31]. Standardization by *R*–*R* interval was performed in both measures to account for the assumption that individuals with higher heart rates are exposed to more pulsatile stress over time given similar pulsatile expansion in each heart cycle. All measurements were made bilaterally. For each segment, the mean PI and FVP of the right and left sides was used. If a pulsatility measure was missing for a paired artery, the measure of the contralateral artery was used.

### Statistical analysis

Statistical analysis was performed in SPSS 24 (IBM Corp, Armonk, NY). Values are mean (SD) if not otherwise specified. Correlations between continuous variables were assessed using Pearson correlation and Spearman’s rho as appropriate. Relationships between categorical variables were assessed using Fisher’s exact test. Relationships between categorical and continuous variables were examined using independent samples *t* test or Kruskal–Wallis *H*-test. Multivariable analysis was performed using generalized linear modeling (GLM). For modeling skewed data such as WMH volume, gamma distribution with log link was used. For normally distributed dependent variables, we used linear distribution with identity link. To limit the number of comparisons, M3 pulsatility was not used in the multivariable analysis. Pulsatility variables were entered one at a time. Three GLM models were thus created, using WMH volume, TBV, and cognitive function as dependent variables, adjusting for age, sex, MAP, hypertension, and tCBF (basic model).

In a second step, the models including all patients (WMH volume, TBV) were additionally adjusted for antihypertensive drugs (by class). The model for cognitive function was additionally adjusted for WMH volume and TBV. A subgroup analysis was performed in patients with SVD. This analysis was also performed using the basic model and used WMH volume and TBV as dependent variables. Patients having completed the neuropsychological examination were deemed too few to use cognitive function as a variable in this analysis. Finally, as periventricular and deep WMH may have different pathogenesis, an ordinal logistic regression model was created to assess these separately using the periventricular and deep scores on the Fazekas scale as dependent variables. This model also adjusted for age, sex, MAP, hypertension, and tCBF. *α* was set at 0.05.

## Results

Of 89 included patients, 64 (71.9%) were diagnosed with ischemic stroke, remaining 25 (28.1%) had TIA (Table 1). Associated SVD was diagnosed in 41 (46.1%) patients. At the time of MRI 71 (79.8%) were treated with one or more antihypertensive agents. Mean age was 70.2 (8.9) years. PI was 1.26 (0.37), 1.28 (0.37), and 1.4 (0.51) in ICA, M1, and M3 respectively. In corresponding segments, FVP was 0.47 (0.15), 0.28 (0.11), and 0.06 (0.02) ml.

**Table 1:** Demographic data

	Small vessel disease^*^	Non-small vessel disease
*N* (%)	41 (46.1)	48 (53.9)
Age, years (SD)	72.1 (9.2)	68.5 (8.3)
Women, *N* (%)	15 (36.6)	12 (25.0)
Stroke, *N* (%)	31 (75.6)	33 (68.8)
NIHSS, median (IQR)	1 (0–2)	0 (0–1)
Etiology, *N* (%)		
Large artery atherosclerosis	11 (26.8)	15 (31.3)
Lacunar	12 (29.6)	–
Cardio-aortic embolus	7 (17.1)	5 (10.4)
Other	1 (2.4)	2 (4.2)
Cryptogenic	10 (24.4)^†^	26 (54.2)^†^
Location, *N* (%)		
Cortical	5 (12.2)	10 (20.8)
Subcortical	12 (29.3)^†^	3 (6.3)^†^
Mixed cortical/subcortical	7 (17.1)	11 (22.9)
Infratentorial	6 (14.6)	4 (8.3)
No lesion	11 (26.8)	20 (41.7)
Risk factors, *N* (%)		
Hypertension	34 (82.9)	43 (89.6)
Hyperlipidemia	35 (87.5)	44 (91.7)
Diabetes mellitus	6 (14.6)	7 (14.6)
Previous cerebrovascular disease	5 (12.2)	5 (10.4)
Ischemic heart disease	8 (19.5)	10 (20.8)
Peripheral artery disease	2 (4.9)	1 (2.2)
Smoking	22 (53.7)	28 (62.2)
Antihypertensive treatment, *N* (%)		
Beta blockers	18 (37.5)	17 (41.5)
RAAS inhibitors	31 (64.6)	26 (63.4)
Calcium channel blockers	20 (41.7)	12 (29.3)
Diuretics	14 (29.2)	15 (36.6)
Blood pressure, mean (SD)		
Systolic	148.0 (21.6)	146.8 (18.2)
Diastolic	78.5 (9.5)	78.4 (10.9)
Mean arterial pressure	101.7 (12.4)	101.2 (11.8)
WMH, mean (SD)		
Fazekas score, PVWM	1.88 (0.87)^†^	0.79 (0.71)^†^
Fazekas score, DWM	1.63 (0.86)^†^	0.71 (0.54)^†^
Fazekas score, total	3.51 (1.60)^†^	1.50 (1.11)^†^
Volume, % ICV, median (IQR)	0.64 (0.17–1.35)^†^	0.11 (0.05–0.24)^†^
TBV, mean (SD)		
Volume, % ICV	64.6 (6.7)^†^	68.1 (5.1)^†^

*NIHSS* National Institutes of Health Stroke Scale, *WMH* white matter hyperintensities, *PVWM* periventricular white matter, *DWM* deep white matter, *ICV* intracranial volume, *TBV* total brain volume

^*^Defined as presence of either total Fazekas score ≥ 4, small recent cortical infarct, lacune, or   >2 microbleeds

†*p* < 0.05

ICA-PI, M1-PI, M3-PI, and ICA-FVP were associated with age (*R* = 0.43, 0.46, 0.34, 0.22; *p* =  < 0.001, < 0.001, 0.002, and 0.044 respectively) and ICA-FVP was associated with decreasing DBP (*R* = − 0.23, *p* = 0.03). There were no significant associations between PI and DBP (ICA-PI: *R* = − 0.07, *p* = 0.5). Women had higher ICA-PI (1.42 vs. 1.19; *p* = 0.03) and M1-PI (1.47 vs. 1.2; *p* = 0.01). This difference remained significant after adjusting for age. PI and FVP were correlated in ICA and M1 (*R* = 0.41, 0.42; both *p* < 0.001) but not in M3.

In the multivariable models, ICA-FVP was associated with WMH volume (*β* = 1.67, 95% CI: [0.1, 3.24], *p* = 0.037) while M1-FVP and both measures of PI were not. If antihypertensives were added to the model, both ICA-FVP and M1-FVP were associated with WMH volume (*β* = 1.91, 95% CI: [0.3, 3.53], *p* = 0.02 and *β* = 2.65, 95% CI: [0.6, 4.7], *p* = 0.011). There was no association between arterial pulsatility and either periventricular- or deep WMH as represented by their respective Fazekas score.

There were no associations between any measure of arterial pulsatility and TBV. Results were unchanged if antihypertensives were added. M1-PI and M1-FVP were associated with decreasing cognitive function (*β* = − 4.4, 95% CI: [− 7.7, − 1.1], *p* = 0.009 and *β* = − 13.15, 95% CI: [− 24.26, − 2.04], *p* = 0.02 respectively). If WMH volume and TBV were added to the model M1-PI, remained associated with decreased cognitive function (*β* = − 4.61, 95% CI: [− 8.01, − 1.21], *p* = 0.008), while M1-FVP did not. The best-fitting basic model for each outcome is presented in Table 2, where other significant co-variates are also displayed.

**Table 2:** Regression analysis

Outcome	Significant predictors	*β*	95% CI	*p*
WMH, % ICV*	**ICA-FVP**	**1.67**	**[0.1, 3.24]**	**0.037**
	Age	0.06	[0.03, 0.09]	< 0.001
	Hypertension	0.83	[0.26, 1.4]	0.005
	TCBF	− 0.006	[− 0.009, − 0.004]	< 0.001
TBV, % ICV^‡^	Age	− 0.34	[− 0.47, − 0.22]	< 0.001
	Female sex	4.17	[2.17, 6.18]	< 0.001
	MAP	0.09	[0.01, 0.17]	0.025
	TCBF	0.02	[0.006, 0.03]	0.003
Cognitive function^‡^ (*n* = 48)	**M1-PI**	− **4.4**	**[**− **7.7, **− **1.11]**	**0.009**
	Age	− 0.33	[− 0.47, − 0.2]	< 0.001
	Female sex	3.84	[1.02, 6.67]	0.008
	MAP	− 0.13	[− 0.22, − 0.04]	0.006

Independent predictors of outcomes from generalized linear models using all patients. Values represent beta coefficients (95% CI) from the overall best-fitting model for each outcome. Model adjusted for age, sex, MAP, hypertension, and tCBF. Measures of cerebral arterial pulsatility in bold

*WMH* white matter hyperintensities, *TBV* total brain volume, *ICV* Intracranial volume, *TCBF* total cerebral blood flow, *FVP* flow volume pulsatility, *MAP* mean arterial pressure

*Gamma distribution, log link

^‡^Linear distribution, identity link

In the SVD subgroup, ICA-FVP was independently associated with WMH volume (*β* = 2.33, 95% CI: [0.3, 4.36], *p* = 0.024). In addition, both ICA-FVP and M1-FVP were associated with decreasing TBV (*β* = − 14.58, 95% CI: [− 25.28, − 3.89], *p* = 0.008; *β* = − 18.72, 95% CI: [− 33.57, − 3.87], *p* = 0.013). PI was not associated with either WMH or TBV. Table 3 presents significant variables from the best-fitting model for each outcome in the SVD subgroup analysis.

**Table 3:** Regression analysis in patients with small vessel disease

Outcome	Significant predictors	*β*	95% CI	*p*
WMH, % ICV^*^	**ICA-FVP**	**2.33**	**[0.3, 4.36]**	**0.024**
	Age	0.04	[0.008, 0.08]	0.016
	Hypertension	0.81	[0.13, 1.49]	0.019
	TCBF	− 0.007	[− 0.01, − 0.003]	< 0.001
TBV, % ICV^‡^	**ICA-FVP**	− **14.58**	**[**− **25.28, **− **3.88]**	**0.008**
	Age	− 0.3	[− 0.48, − 0.12]	0.001
	MAP	0.16	[0.04, 0.27]	0.009
	TCBF	0.03	[0.006, 0.05]	0.009

Independent predictors of outcomes from generalized linear models in patients with small vessel disease. Values represent beta coefficients (95% CI) from the overall best-fitting model for each outcome. Model adjusted for age, sex, MAP, hypertension, and tCBF. Measures of cerebral arterial pulsatility in bold

*WMH* white matter hyperintensities, *TBV* total brain volume, *ICV* intracranial volume, *TCBF* total cerebral blood flow, *FVP* flow volume pulsatility, *MAP* mean arterial pressure

*Gamma distribution, log link

^‡^Linear distribution, identity link

M1-PI was increased in patients with SVD (1.37 vs. 1.2, *t* test, *p* = 0.024). This difference was no longer significant after adjusting for age. There was no difference in pulsatility between stroke versus TIA or between different etiologies or lesion locations. There were no correlations between NIHSS score and arterial pulsatility.

## Discussion

Using a novel MR technique in stroke patients, arterial pulsatility was measured and correlated to typical features of small vessel disease (SVD). Increased FVP, a new parameter measuring cyclic expansions of the vascular tree, was associated with increased WMH volume, decreased cognitive function and, in patients with associated SVD, lower TBV. The most commonly used parameter for arterial pulsatility, PI, was only associated with cognitive function.

4D flow MRI is a relatively new technique for measuring intracranial flow. We have previously shown that 4D flow measurements are consistent with traditional phase-contrast MRI in both young and elderly subjects, and that good agreement between the modalities also applies to pulsatility measures [26, 32]. PI and FVP are different parameters, both describing arterial pulsatility. In our data, FVP had stronger associations with features of SVD compared to PI. In a wider perspective, PCMRI studies tend to report weaker associations between arterial PI and SVD compared to TCD dittos [11, 14–16, 33, 34]. This may be due to the inferior time resolution of PCMRI and 4D flow MRI causing the systolic peak to be blunted, thus underestimating PI in subjects with high pulsatility. Furthermore, waveform peak to peak detection needed for PI need a good signal-to-noise ratio (SNR). FVP is based on accumulated flow and estimates the volumetric expansion of the arterial tree distal to the measuring point. This means it is less sensitive to limited time resolution or SNR. It may also be argued that this approach is more physiological than the more commonly used parameter PI. We have previously established that FVP is a reliable biomarker for pulsatile flow in arteries, veins, and CSF using phase-contrast MRI [13]. A caveat, however, is that it tends to decrease linearly with time in the scanner. If possible, it is thus preferable to standardize the MRI protocol. 4D flow MRI estimates of FVP are highly consistent with traditional phase-contrast estimates [26]. Based on this, we propose that FVP may be a useful biomarker for assessing pulsatile stress with PCMRI and 4D flow MRI. To our knowledge, this is the first study to evaluate the relationship between FVP and both WMH and cognitive function. We have previously described a relationship between FVP and regional brain volumes [30]. Further studies are needed to evaluate the usefulness of FVP as a biomarker of cerebral arterial pulsatility.

There was an association between FVP and WMH. This is a novel finding, as FVP has not been measured in this context previously. There is steadily increasing evidence of a relationship between WMH and high cerebral pulsatility, spanning a variety of modalities, anatomical measuring points, and populations. In a retrospective, hospital-based cohort of 450 patients, baseline M1-PI was found to predict WMH expansion independent of traditional risk factors [35]. In patients with stroke and TIA, two studies (both *n* = 100) found cross-sectional relationships between M1-PI and WMH [14, 15]. Our data add to this evidence by means of a more physiological approach to cerebral pulsatility as well as a novel modality.

We also found a negative correlation between arterial pulsatility measured in the acute phase, and cognitive impairment 1 year later. This relationship is sparsely studied, but our results are consistent with previous studies in the elderly population and in patients with acute lacunar syndromes [12, 17]. The relationship remained significant after adjusting for WMH volume and TBV implying a mechanism separate to macrostructural change. Indeed, there are some data to support a relationship between increasing pulsatility and loss of integrity in normal appearing white matter [33] which, in turn, could present as cognitive impairment. A relationship between increased cerebral pulsatility and cognitive impairment are further supported by longitudinal evidence from studies of arterial stiffness. Several prospective cohorts have found aortic stiffness to be an independent risk factor for cognitive decline, cognitive impairment, and dementia [36–39]. The pathophysiological pathway of the relationship between aortic stiffness and cognitive impairment has not been established, but increased transfer of blood flow pulsatility is a generally agreed-upon hypothesis in the literature [36, 37, 39]. Our findings of an association between direct measurements of cerebral pulsatility and cognitive function further support this pathway.

In stroke patients with associated SVD, we found a negative association between FVP and TBV. This correlation was not significant in the full sample, nor using pulsatile index. This relationship has not been previously studied in patients with stroke, but data from the AGES-Reykjavik population study and a previous study in elderly from our group support a relationship between cerebral arterial pulsatility and brain volume [12, 30]. The existence of a dose–response relationship between pulsatility and TBV only in patients with associated SVD can in our study not be explained by differences in pulsatility or risk factors. However, patients with SVD trended towards higher age, which may reflect duration of pulsatility exposure.

Several mechanisms have been put forward to explain the relationship between cerebral arterial pulsatility and brain structure and function. In one, increased aortic stiffness and impedance-matching with the carotid arteries enables transfer of increased arterial pulsatility into the cerebral microcirculation [12, 14]. An important task for cerebral arterioles is regulating flow to downstream capillaries, providing the correct volume to meet current metabolic demand and at the same time protecting capillaries from exposure to excessive pressure. Increased flow pulsatility has been proposed to cause hypertrophic remodeling and lumen narrowing in these vessels [6, 7]. Indeed, both increased large vessel pulsatility and increased stiffness of penetrating arterioles predicted WMH expansion in a recent publication [35]. Our data support this hypothesis by showing increased mechanical load, expressed as downstream cyclic expansion, is correlated to WMH, cognitive impairment, and in patients with SVD, TBV.

Cerebral veins and venules have been less explored in the setting of SVD. There is some evidence to suggest venous collagenosis may be associated with WMH [40, 41]. The pathophysiology behind this finding is unknown, but one suggestion is that venous collagenosis is secondary to arterial disease [42]. Increased pulsatility could be a link between arterial and venous disease as kinetic energy may be transferred from arteries to veins by way of capillaries and/or CSF, and increased venous pulsatility has been tied to increasing WMH [16]. However, a possible association between venous pulsatility and venous vascular pathology has to our knowledge not been studied.

It has previously been assumed that increased WMH causes increased cerebral arterial pulsatility by way of increased flow resistance. However, several studies have found that cerebral arterial pulsatility is more closely associated with upstream factors such as aortic stiffness, aortic pressures, and impedance-matching at the aortic–carotid junction [12, 14]. This supports cerebral arterial pulsatility as a causative factor in WMH rather than solely an effect.

Strengths of the study include prospective recruitment, use of FVP in addition to PI, performing a comprehensive, validated neuropsychological test battery, for the first time assessing the relationship of pulsatility and atrophy in this population and performing the MRI examination in a clinically relevant situation, within the initial hospitalization. Moreover, 4D flow MRI is feasible to perform as part in routine management of stroke patients as measurements are made entirely in post-processing and can be automatized [43, 44].

This study also has limitations. As WMH, TBV, cognitive function, hypertension, and cerebral arterial pulsatility are all closely associated with age, causative associations are difficult to find. This particularly applies to wide-range populations and cross-sectional studies such as this. This implies that relevant associations between these variables may be underestimated. The sample size, although similar to previous studies in the field, was insufficient to detect weaker correlations. Patients with severe hemodynamic alterations (SBP > 180 mmHg, CAS > 70%) were excluded, as measured pulsatility was not deemed representative of their long-term pulsatility exposure. Results may, therefore, not be generalizable to these patients. This study did not account for venous pulsatility, which also may be associated with WMH in stroke patients [16], somewhat limiting conclusions regarding pathophysiology. MRIs were performed within days of the cerebrovascular event. It is currently unknown how an acute cerebrovascular event affects pulsatility, although there are data to suggest that pulsatility may be altered in the acute phase compared to baseline [15]. This may lead to underestimation of the strength of associations between long-term pulsatility and outcomes in our data. Potential effects of antihypertensive treatments on cerebral arterial pulsatility are also unknown and likely heterogeneous depending on mechanisms of action. Neuropsychological tests were performed approximately 1 year after the acute event to avoid acute, reversible cognitive symptoms. The time passed between measurements of pulsatility and cognitive function may cause underestimation of this relationship. Additionally, only 54% of patients participated in the neuropsychological follow-up. We endeavored to quantify brain volumes in an as objective and automatized fashion as possible. When segmenting WMH, however, manual correction was needed to avoid including acute infarctions and certain extracerebral features such as the cerebral falx and transverse sinus. Corrections were restricted to the aforementioned features and the operator was blinded to pulsatility data.

## Conclusions

New and clinically relevant findings reported in this study are associations between the novel parameter FVP and both WMH and cognitive function. FVP was more strongly associated with features of SVD compared to PI and may be a useful biomarker in future studies of cerebral pulsatility. The cross-sectional design limits conclusions regarding causality. However, our results support an association between arterial pulsatility and SVD in stroke patients, and provides a potential target for further research and preventative treatment. This study also shows that 4D flow MRI could be a suitable method for flow assessment in clinical routine and in large cohorts.
